# Crimean–Congo Hemorrhagic Fever Virus Survey in Humans, Ticks, and Livestock in Agnam (Northeastern Senegal) from February 2021 to March 2022

**DOI:** 10.3390/tropicalmed7100324

**Published:** 2022-10-21

**Authors:** Moufid Mhamadi, Aminata Badji, Idrissa Dieng, Alioune Gaye, El Hadji Ndiaye, Mignane Ndiaye, Moundhir Mhamadi, Cheikh Talibouya Touré, Mouhamed Rassoul Mbaye, Mamadou Aliou Barry, Oumar Ndiaye, Babacar Faye, Fatimata Amadou Ba, Boly Diop, Mamadou Ndiaye, Mathioro Fall, Samba Niang Sagne, Gamou Fall, Cheikh Loucoubar, Hugues Fausther-Bovendo, Amadou Alpha Sall, Gary Kobinger, Ousmane Faye, Mawlouth Diallo, Oumar Faye

**Affiliations:** 1 Institut Pasteur de Dakar, Virology Department, Dakar 12900, Senegal; 2 Parasitology Department, Université Cheikh Anta Diop de Dakar, Dakar 10700, Senegal; 3 Institut Pasteur de Dakar, Medical Zoology Department, Dakar 12900, Senegal; 4 Institut Pasteur de Dakar, DIATROPIX, Dakar 12900, Senegal; 5 Institut Pasteur de Dakar, Epidemiology, Clinical Research and Data Science Department, Dakar 12900, Senegal; 6 Microbiology, Immunology and Infectious Pathology Service, Department of Public Health and Environment, EISMV of Dakar, Dakar 12900, Senegal; 7 Ministry of Health and Social Action, Dakar 12900, Senegal; 8 Ministry of Livestock and Animal Production, Dakar 12900, Senegal; 9 Global Urgent and Advanced Research and Development, Batiscan, QC G0X 1A0, Canada; 10 University of Texas Medical Branch, Galveston, TX 77555-0132, USA

**Keywords:** CCHF, prevalence, human, sheep, tick

## Abstract

Crimean–Congo hemorrhagic fever virus (CCHFV) is widespread in Asia, Europe, and Africa. In Senegal, sporadic cases of CCHFV have been reported since 1960. Bordering Mauritania in northeastern Senegal, Agnam is an arid area in the region of Matam where CCHFV is endemic, which harbors a pastoralist community. Given the drought conditions of Agnam, inhabitants are in constant movement with their animals in search of pasture, which brings them into contact with pathogens such as arboviruses. To identify CCHFV in this area, we established a One Health site in order to analyze animal livestock, ticks and human samples collected over a one-year period by qRT-PCR and ELISA. Our analysis showed one (1/364) patient carried anti-CCHFV IgM and thirty-seven carried anti-CCHFV IgG (37/364). In livestock, anti-CCHFV IgG was detected in 13 (38.24%) of 34 sentinel sheep. The risk of CCHFV infection increased significatively with age in humans (*p*-value = 0.00117) and sheep (*p*-value = 1.18 × 10^−11^). Additional risk factors for CCHFV infection in sheep were dry seasons (*p*-value = 0.004) and time of exposure (*p*-value = 0.007). Furthermore, we detected a total of three samples with CCHFV RNA within *Rhipicephalus evertsi evertsi* and *Rhipicephalus guilhoni* tick species. Our results highlighted the usefulness of a One Health survey of CCHFV in pastoral communities at risk of arboviruses.

## 1. Introduction

Crimean–Congo hemorrhagic fever virus (CCHFV) is a tick-borne virus mainly transmitted by ixodid (hard-bodied) tick bites. CCHFV belongs to the *Bunyavirales* order and is a member of the *Orthonairovirus* genus [1]. As with all *Bunyavirus*, the CCHFV genome is a three-segment negative-strand RNA, composed of small (S), medium (M), and large (L) segments. The S segment encodes the nucleocapsid (N) protein and nonstructural proteins (NSs), which are translated from overlapping open reading frames. The M segment encodes for glycoproteins (Gn and Gc) and a nonstructural protein (NSm), while the L segment encodes for the RNA-dependent RNA polymerase [2].

Tick bites remain the principal route of CCHFV transmission in humans and animals. However, the infection in humans could occur also after the butchering of infected livestock and in the healthcare setting during the care of infected patients [2]. Animals are asymptomatic reservoirs of the virus, but infection in humans, after a few days of incubation, can cause nonspecific febrile illness characterized by fever, myalgia, diarrhea, nausea, and vomiting. In a few cases, symptoms have progressed to severe hemorrhagic disease. The case fatality rates range from <5% to upwards of 30% [3]. Infection kinetics studies show viral RNA can be detected in blood by RT-PCR for up to 18 days of the illness [4]. IgM antibody response is detectable by day 7, and IgG antibody response is detectable by day 7–9 of illness. The highest of IgM and IgG antibody titers are usually attained from days 14 to 21 of infection. The increase of antibody response seems to coincide with the decline of viremia, which is supported by the diminished viral RNA in surviving patients, suggesting this antibody response plays a role in viral elimination. By 4–6 months after illness, the IgM antibodies become undetectable, whereas the IgG titers remain detectable for at least 5 years post infection [4].

No specific treatment or vaccine is approved for CCHFV. However, ribavirin has been proven to work if used in the first days of the onset of symptoms [5,6]. 

First reported in 1944 in the Crimea region of the Soviet Union, and later in 1956 in the Belgian Congo, CCHFV has been reported in more than 30 countries in Africa, Europe, and Asia [3,7]. The near global spread of CCHFV may have been facilitated by migratory birds infested with CCHFV-infected ticks [8]. *Hyalomma*, *Rhipicephalus*, and *Amblyomma* ticks have been proven competent to transmit the disease [9,10].

In West Africa, recurrent CCHFV outbreaks have been reported [11,12], with serological and molecular evidence of CCHFV circulation reported in Senegal since 1960 [13]. Due to their climate (mostly hot and dry), geographical positions (trans-frontal with endemic countries such as Mali and Mauritania), and pastoral activity [14,15], the northern Senegal regions have populations that are at high risk for a CCHFV outbreak. Additionally, the most recent human CCHFV case was reported in Bokidiawe, located in Matam, a north Senegal area [16]. To surveil and conduct research on arboviruses and viral hemorrhagic fever in northern Senegal, the World Health Organization collaborating centre (WHOCC) collaborated with the Virology Department and the Medical Zoology Department of Institut Pasteur de Dakar (IPD) to establish a One Health site in Agnam (16°00′18″ N, 13°41′35″ W) on the border of Mauritania. In this study, a CCHFV One Health survey on humans, sheep, and ticks was conducted in Agnam, a north Senegal area at high risk of CCHFV emergence. Through this survey, evidence of the circulation of CCHFV was found in humans, livestock, and ticks. 

## 2. Materials and Methods

### 2.1. Study Area

**Figure 1:**
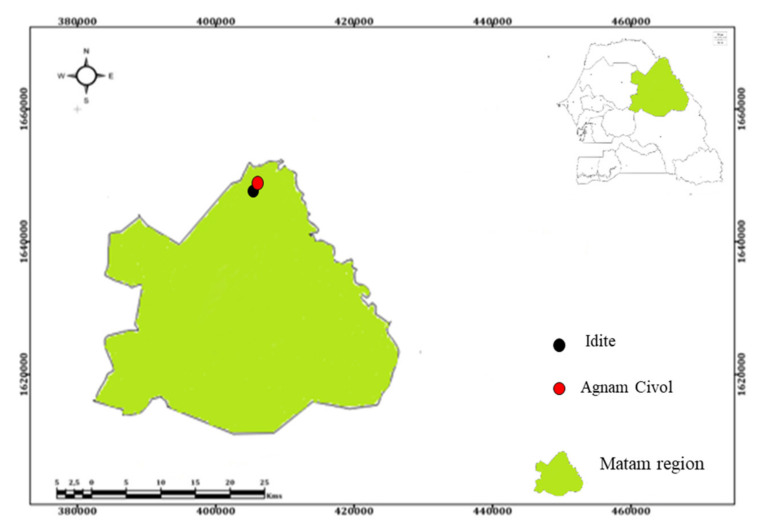
Study Areas: Agnam Civol health care center (represented by the red dot) for human sampling and Idite (represented by the black dot) for sheep and tick sampling. These areas are 14.8 km apart and are located in the Agnam district in the region of Matam (colored in green) in the Northern Senegal.

In February 2021, a One Health site was established in Agnam (16°00′18″ N, 13°41′35″ W) in northern Senegal, near the border of Mauritania. The rainy season in Agnam lasts four to five months (June, July, August, September, and October), with the average rainfall at less than 369 mm/year. Here, pastoralism is highly practiced, and our study areas included sheep and tick sampling a mere 14.8 km from the health care center where human samples were collected (Figure 1).

### 2.2. Blood Sample Collection of Humans and Livestock

Venous blood had been taken from every patient with febrile syndrome in the Agnam health care sentinel site since June 2021. Every patient enrolled in the survey provided informed consent. Consent for minors was provided by a parent or guardian. For inclusion, the patient needed to have a temperature above or equal to 38 degrees with two minor symptoms (headache, myalgia, arthralgia, retro-orbital pain) or with one major sign of hemorrhagic manifestation (purpura, epistaxis, gingivorrhagia, metrorrhagia). Clinical and social/demographic data such as living address, age, travel history, and vaccination history were collected. Blood samples were sent weekly to Institut Pasteur to diagnose arboviral and viral hemorrhagic fever infection.

For the livestock survey, 34 sheep were selected from villages located in the study area, in February 2021. These sheep were bled by jugular routes and tested for current and previous signs of CCHFV infection by qRT-PCR and ELISA. After inclusion, seronegative sheep were blood sampled every 14 days for 56 days, then monthly to monitor CCHFV infection until March 2022.

### 2.3. Ticks Sampling

A sampling of ticks from animals was undertaken at the Agnam sites where the sheep were sampled. Sheep were physically restrained and the whole body was visually examined for ticks. If found, the ticks were pulled off manually, placed in a sterile tube, and transported to the laboratory in liquid nitrogen. In the laboratory, ticks were washed in sterile water, ethanol, and an L15 medium containing antimicrobial agents (100 U/mL penicillin, 100 μg/mL streptomycin, and 1 μL/mL amphotericin B). Ticks were identified per species by using taxonomic keys and pooled in groups of 1 to 28 by species, sex, collection date, and site. The tick pools were homogenized by using sterile mortar and pestle with 0.5 mL–2 mL ice-cold L15 medium (10% Fetal Bovine Serum, 100 U/mL penicillin, 100 μg/mL streptomycin, and 1 μL/mL amphotericin B) under high containment. The homogenates were clarified by centrifugation at 2500 rpm for 5 min at 4 °C. Then, supernatants were stored at −80 °C until use.

### 2.4. Serological Assay for CCHF

Human sera were screened for anti-CCHFV IgM by immunocapture ELISA [17]. For this purpose, plates were coated by adding goat anti-human IgM in a carbonate-bicarbonate buffer (0.015 M sodium carbonate, 0.035 M sodium bicarbonate, pH 9.6) and incubated at 4 °C overnight. After washing with washing buffer (PBS1x +Tween 0.05%), samples were added and the plates were incubated at 37 °C for one hour. After incubation, plates were washed, then mouse brain CCHFV antigen was added and plates were incubated at 37 °C for one hour. Plates were washed and specific CCHFV hyperimmune mouse ascite was added, then the plates were incubated at 37 °C for one hour. An anti-mouse IgG antibody conjugated with horseradish peroxidase was added after washing, then the plates were incubated at 37 °C for one hour. Tetramethylbenzidine (TMB) was added after washing, then sulfuric acid was used for blocking the reaction. Optical densities (OD) were read using a reader with 450/620 filters and the data were processed in Excel. The OD value 0.2 was used as a cutoff. 

Anti-CCHFV IgG presence was tested in both animal and human sera. For this, CCHFV recombinant glycoprotein Gn (Sinobiological) was diluted at 50 ng in PBS1x (Sigma-Aldrich) and added in plates, then these plates were incubated overnight at 4 °C for coating. After washing with washing buffer (PBS1x +Tween 0.05%), the antigen residues were captured with blocking buffer (PBS1x +Tween 0.05% + 5% skimmed milk). After 1 h of incubation at 37 °C, the plates were washed, 1/100 diluted sera were added, and the plates were incubated. After washing, a specific antibody conjugated with horseradish peroxidase (rabbit anti-sheep IgG (Biorad) diluted at 1/20,000 or a goat anti-human IgG (KPL) diluted at 1/10,000), then the plates were incubated for 1 h at 37 °C. TMB was added and then the reaction was blocked with sulfuric acid. Optical densities (OD) were read using with 450/620 filters and the data were processed in Excel. The cutoff was determined with a finite mixture model in R software.

### 2.5. qRT-PCR for CCHFV

RNA was extracted from human and sheep sera and tick supernatant using the QIAamp RNA Viral Kit (Qiagen GmbH, Heiden, Germany) according to the manufacturer’s recommendations. RNA was eluted in 60 μL of AVE buffer and stored at −80 °C until use.

CCHFV virus detection was performed using the AgPath-ID One-Step RT-PCR kit Thermofisher© (Waltham, Massachusetts, USA) and primers and probe as previously described [18]. A total of 5 μL of RNA was added to 20 μL of reaction mixture consisting of 12.5 μL of buffer, 4 μL of RNase free water, 1 μL of each primer, 0.5 μL of probe, and 1 μL of enzymes. The qRT-PCR was performed on QuantStudio 5 (Applied Biosystems, Foster City, CA, USA). The cycling conditions were 50.0 °C for 10 min, at 95.0 °C for 15 min, and 40 cycles of 15 s at 95.0 °C, and 1 min at 60 °C.

### 2.6. Statistical Analysis

Statistical analysis was performed on R software version 4.1.3 using chi-squared/Fisher’s exact test for qualitative analysis, and logistic regression was used to evaluate the risk of CCHFV infection. Statistical significance was defined as *p* < 0.05.

The force of infection was determined on R software for both humans and animals using the single serosurvey method and the repeated serosurvey method, respectively, as previously described [19].

## 3. Results

### 3.1. Human Survey

From June 2021 to March 2022, 364 human sera were collected from febrile patients with a median temperature of 38.5 °C. Gender proportion was near equal for this population (46.7% male and 53.3% female) (X^2^ = 0.6272, df = 1, *p* = 0.4284). The most reported signs and symptoms were headache (96.97%) followed by myalgia (76.64%), arthralgia (18.68%), vomiting (4.12%), hemorrhage (0.82%), and asthenia (0.27%). Patient ages were distributed between 2 months and 98 years, with a median age of 20 years. The most reported age ranges were 10–20 years (14.63%), followed by 20–30 years (30.75%) (X^2^ = 103.39, df = 9, *p* < 2.2 × 10^−16^), then 30–40 years (22.39%). Most patients came from Agnam areas such as Agnam Civol (49.73%) and Agnam Ouro Ciré (17.31%). Bélé and Fetediabe were the most sampled areas (4.39%) besides the Agnam areas (X^2^ = 319.95, df = 13, *p* < 2.2 × 10^−16^) (Table 1). The majority of febrile patients were sampled in October (22.29%), then November (14.29%), December (12.09%), and January (10.99%) (X^2^ = 29.684, df = 9, *p*-value = 0.0004964). No difference was noticed between the proportions of patients sampled during the dry season (54.12%) and the rainy seasons (45.88%) (X^2^ = 0.67927, df = 1, *p*-value = 0.4098) (Table 1).

Anti-CCHFV IgM detection assay reveals that one patient was positive, while thirty-seven were positive for anti-CCHFV IgG, and all samples were negative by qRT-PCR. The IgM positive patient, a 10-year-old child, sought consultation 3 days after the onset of symptoms. His clinical signs and symptoms included fever (39.4 °C), headache, and joint pains. This IgM-positive patient was negative to an IgG test at this timepoint, but became positive to the IgG test 20 days later. The patient ultimately recovered without complications. The median age of the CCHFV seropositive (IgM+IgG) patients was 31 years, and though females out-represented males, no significant difference was noticed [OR = 0.67 (0.32, 1.33) and *p* = 0.257]. The risk of CCHFV exposure increased by 1.03 years in our sampling areas. Within our sampling groups, months, seasons, and location were not risk factors for CCHFV exposure (Table 2). Globally, the seroprevalence was 10.44% in this area.

**Figure 2:**
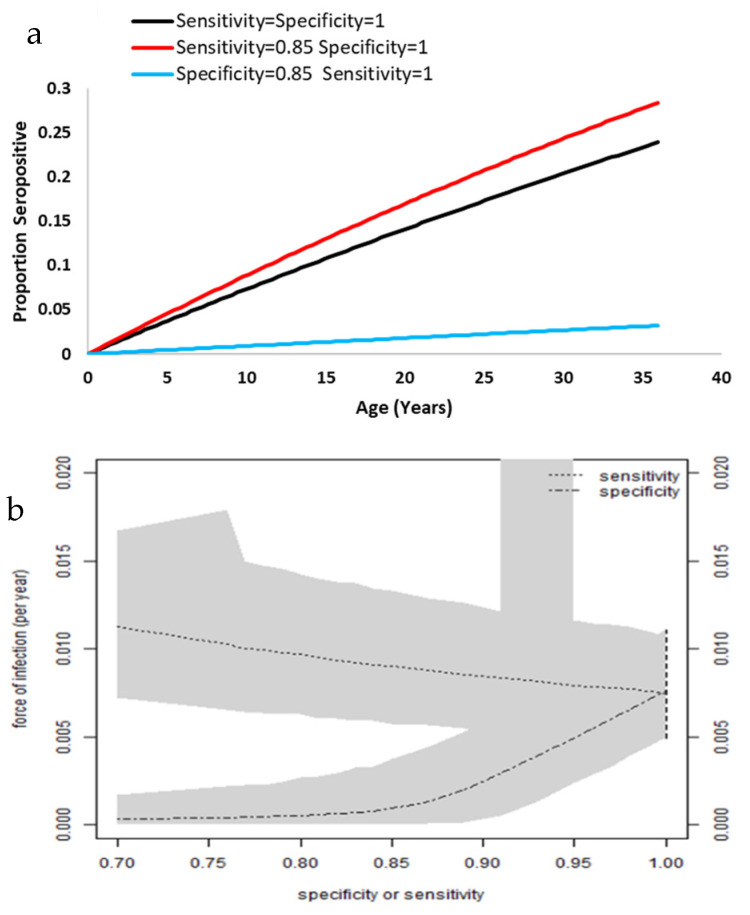
Representation of seropositivity by age and the force of infection. (**a**) Proportion of seropositive patients by age. (**b**) Relation between force of infection, sensitivity, and specificity. The grey zones represent the 95% credible intervals. As the credible intervals approach the 95% confidence interval (vertical dashed line), the sensitivity and specificity approach 100%.

The fitted proportions of seropositive patients by age are shown in Figure 2a. The values of 85% sensitivity and specificity have been chosen to illustrate the method. We found that imperfect specificity induces low seroprevalence and imperfect sensitivity suggested higher seroprevalence. Analysis estimated a force of infection at 7.6% per year (CI: 4.9–11.1%) (Figure 2b).

### 3.2. Sheep Survey

**Figure 3:**
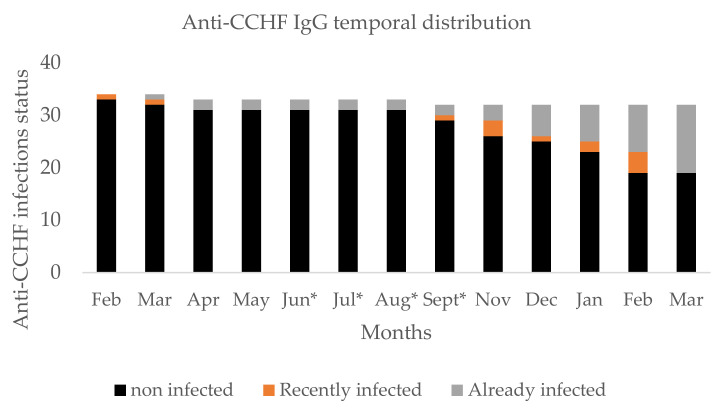
Temporal distribution of anti-CCHFV antibody in livestock sheep. Legend: * on month indicates the rainy seasons.

From February 2021 to March 2022, 453 sera were sampled from our 34 sentinel sheep. At the beginning of the study, this population principally contained females (94.11%) aged between 4 and 18 months with a median age of 8 months. During the survey, two sheep deaths were recorded, but CCHFV infection was not detected by qRT-PCR and ELISA. The global seroconversion rate was 38.24%, and almost all seroconversion occurred in the dry season (Figure 3).

Risk factor analysis shows that adult sheep were more exposed than juveniles and the risk increased by 1.26 months (age) for CCHFV exposure. Sheep were more exposed to CCHFV during the dry season than during the rainy seasons. Furthermore, logistic analysis shows that CCHFV exposure increased significantly in January, February, and March 2022. Sex and tick infestation had no influence on CCHFV positivity during the survey (Table 3). 

**Figure 4:**
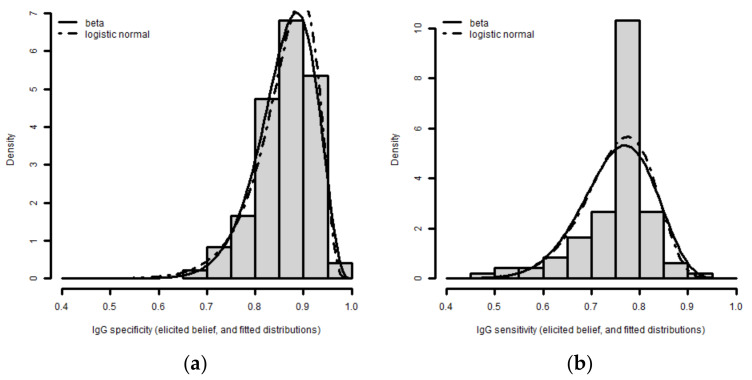
IgG uncertainty in (**a**) specificity and (**b**) sensitivity.

**Figure 5:**
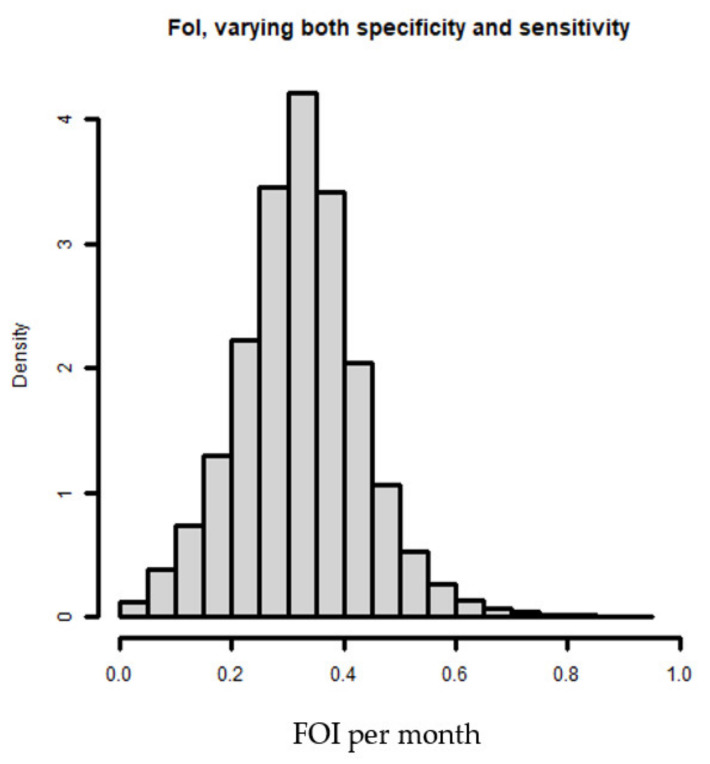
Estimation of the force of infection.

Figure 4 shows the elicited distributions of the Anti-CCHFV IgG ELISA for the sensitivity and specificity. The figure summarizes the beliefs of the three experts by linear pooling [19] and represents the fitted beta and logistic-normal distributions that we used to generate the force of infection (Figure 5). We found that sensitivity presented high variance compared to specificity and the distribution for specificity was near 100% (Figure 4).

The estimated force of infection is 32.14% per month with a credible interval of 10.1 to 55.59%, with sensitivity and specificity both varying (Figure 5).

### 3.3. Ticks

**Figure 6:**
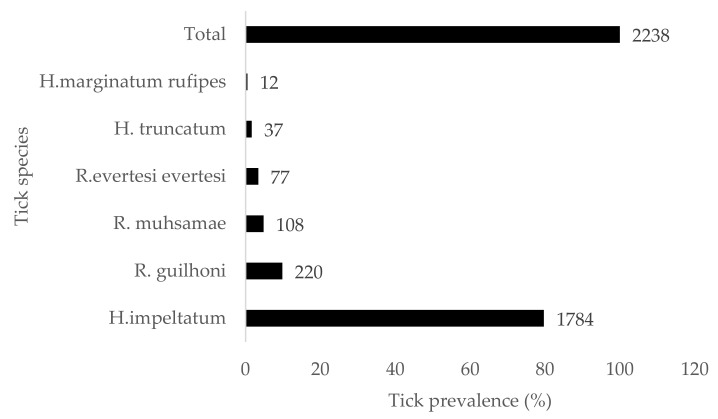
Prevalence of tick species.

During the survey, a total of 2238 ticks of two genera and six species were sampled. *Hyalomma impeltatum* was the most predominant species (79.71%), followed by *Rhipicephalus guilhoni* (9.83%), *R. muhsamae* (4.83%), *R. evertsi evertsi* (3.44%), *H. truncatum* (1.65%), and *H. marginatum rufipes* (0.54%) (Figure 6). CCHFV RNA were detected by qRT-PCR in three (3) samples of ticks among all 808 pools, two of these were from a pool of *R. evertsi evertsi* (Ct = 34.5 and Ct = 22.15) and the other was from *R. guilhoni* (Ct = 28.42).

## 4. Discussion

Among the humans tested in Agnam, one was positive for CCHFV anti-IgM. This patient had contact with cattle, goats, and sheep, which classified him as high-risk. This is the first documented presumptive acute CCHFV case detected in this area. Human overall seroprevalence is 10.44%, similar to the 11.3% reported in Rosso (Senegal) [15], the 13.1% reported in Senegal [14], and the 14% reported in Ijara (Kenya) [20]. Our study showed that age influenced seropositivity. The risk for CCHFV infection in humans increased by 1.03 year, which echoed results found in Kenya [20]. We estimated the force of infection (FOI) at 7.6% per year in Agnam. This estimation of FOI is lower than the 13.7% found previously in a Colombo study [19].

In livestock sheep, 38.24% had seroconverted to CCHFV between February 2021 and March 2022. Our results compare to the 35.3% in India [21], the 32.6% in Kosovo [22], and the 32.8% in the Saint-Louis region of Senegal [23]. Our seroprevalence is higher than the rate reported in Mauritania (16%) [24], and Niger (3%) [25]. As an explanation, unlike our study, the samples of these studies were undertaken in many areas with different epidemiological facets. We noted that 12 of the 13 cases of CCHFV seroconversion among sentinel sheep occurred during the dry season. This result supports epidemiological reports of higher infection rates of CCHFV in Senegal during the dry season [23]. This seasonal increase could be due to the abundance of CCHFV population vectors during the dry season [23]. Analysis shows that seropositivity increases by 1.26 months (age) and increases with time of exposure in natural settings. In natural settings, naïve livestock need at least 6 months before seroconversion to CCHFV. Furthermore, the analysis of CCHFV immunity (IgG) remains possible for at least 3 months. The FOI was 32.14% per month for our livestock sheep, a value higher than the 13.3% found in a Medellin study [19]. It is possible that our population has greater exposure to the disease.

CCHFV screening in ticks showed that three samples contained CCHFV RNA, with two pools of *R. evertesi evertesi* and one of *R. guilhoni* tick species. CCHFV had been found previously in *Rhipicephalus* species in Senegal [26] and Mauritania [27]. Additionally, in Senegal, *R. evertesi evertesi* had been found to be a competent vector for CCHFV transmission [9]. Unlike Ghana [28], Sudan [29], and Kenya [30], CCHFV was not detected in ticks of the *Rhipicephalus* genus, suggesting different evolutionary pressures may be associated with regional vectors. 

Future studies determining the genetic characterization of CCHFV viral RNA in ticks will inform the disease epidemiology in areas of Agnam. 

CCHFV circulation was demonstrated in humans, livestock, and ticks in areas of Agnam. These results highlighted the importance of a One Health approach to prevent and respond to CCHFV outbreaks. Our findings indicate that in Agnam (Senegal, northern area) the peak of transmission occurs in February and March. As such, vaccine candidate trials for CCHFV or a livestock tick control campaign should be implemented before this high-risk transmission period.

## Figures and Tables

**Table 1:** Social and demographical presentation of the human samples.

Criteria	N (%)
Sex	
M	170 (46.70%)
F	194 (53.30%)
**Age range**	
[0–10]	49 (14.63%)
[10–20]	103 (30.75%)
[20–30]	75 (22.39%)
[30–40]	55 (16.42%)
[40–50]	31 (9.25%)
[50–60]	11 (3.28%)
[60–70]	9 (2.69%)
[70–80]	1 (0.30%)
[80–90]	0
[90–100]	1 (0.30%)
**Location**	
Agnam Civol	181 (49.73%)
Agnam Godo	13 (3.57%)
Agnam Goly, Barga, Idite	1 (1.64%)
Agnam Ouro Ciré	63 (17.31%)
Agnam Ouro Mollo, Badiya, Karadji, Ngouloum, Nodi, Orefonde, Thilogne	1 (1.92%)
Agnam Sinthou Cire	12 (3.30%)
Agnam Thiodaye	11 (3.02%)
Asnde Balla	5 (1.37%)
Bagonde	24 (6.59%)
Balanabé	4 (1.10%)
Bele, Fetediabe	8 (4.39%)
Yero Yabe	7 (1.92%)
Toulel Thiale	13 (3.57%)
**Months**	
June	13 (3.57%)
July	5 (1.37%)
August	31 (8.52)
September	36 (9.89%)
October	81 (22.25%)
November	52 (14.29%)
December	44 (12.09)
January	40 (10.99%)
February	33 (9.06%)
March	29 (7.97%)
**Seasons**	
Dry	197 (54.12%)
Rainy	167 (45.87%)

**Table 2:** Risk factors for CCHFV in human populations.

	N IgG (%)	OR (CI, 95%)	*p*-Value
**Gender**			
Man	15 (39.73%)	0.67 (0.32, 1.33)	0.257
Woman	23 (60.52%)		
**Age**			
	NA	1.03 (1.01, 1.05)	0.00117
**Season**			
Dry	25 (65.79%)	0.59 (0.27, 1.18)	0.149
Rainy	13 (34.21%)		
**Months**			
June * 2021	4 (10.52%)	3.00 (6.00, 1.52)	0.172
July * 2021	0	4.31 (2.04, 6.01)	0.989
August * 2021	4 (10.52%)	3.00 (6.00, 1.52)	0.172
September * 2021	1 (2.63%)	1.92 (9.56, 1.39)	0.151
October * 2021	4 (10.52%)	3.50 (7.79, 1.57)	0.157
November 2021	2 (5.26)	2.70 (3.57, 1.47)	0.144
December 2021	2 (5.26)	3.21 (4.24, 1.76)	0.207
January 2022	10 (26.31)	2.25 (6.66, 8.97)	0.211
February 2022	5 (13.16)	1.20 (2.89, 5.32)	0.796
March 2022	5 (13.16)	1.40 (3.34, 6.25)	0.639
**Location**			
Agnam Civol	18 (47.36%)	7.28 (2.56, 1.94)	0.533
Agnam Godo	1 (2.63%)	6.59 (3.45, 3.87)	0.702
Agnam Goly, Barga, Idite	2 (5.26%)	7.90 (2.98, 2.09)	0.154
Agnam Ouro Ciré	6 (15.79%)	8.32 (2.73, 2.31)	0.732
Agnam Ouro Mollo, Badiya, Karadji, Ngouloum, Nodi, Orefonde, Thilogne, Lidoube	1 (2.63%)	6.83 (NA, Inf)	0.998
Agnam Sinthou Cire	0	6.83 (1.56, 1.30)	0.993
Agnam Thiodaye	3 (7.89%)	2.96 (5.85, 1.21)	0.147
Asnde Balla	1 (2.63%)	1.97 (9.64, 1.49)	0.558
Bagonde	4 (10.52%)	1.58 (4.05, 5.18)	0.47
Balanabé	1 (2.63%)	1.63 (2.24, 2.27)	0.418
Bele, Fetediabe	0	6.83 (NA, 7.78)	0.994
Yero Yabe	0	6.83 (NA, 1.59)	0.995
Toulel Thiale	1 (2.63%)	6.83 (2.49, 5.83)	0.993

Legends: * indicate months of the rainy seasons.

**Table 3:** Risk factors for CCHFV in livestock sheep.

	N IgG (%)	OR (CI, 95%)	*p*-Value
**Age**			
Juvenile	0	1.26 (1.18, 1.35)	1.18 × 10^−11^
Adult	13 (100%)		
**Seasons**			
Dry	12 (92.30%)	0.33 (0.15, 0.68)	0.004
Rainy	1 (7.70%)		
**Months**			
February 2021	1 (7.70%)	0.48 (0.02, 5.30)	0.092
March 2021	1 (7.70%)	1 (0.11, 8.75)	0.071
April 2021	0	1 (0.11, 8.75)	1
May 2021	0	1 (0.11, 8.75)	1
June * 2021	0	1 (0.11, 8.75)	1
July * 2021	0	1 (0.11, 8.75)	1
August * 2021	0	1 (0.11, 8.75)	1
September * 2021	1 (7.70%)	1.54 (0.24, 12.36)	0.644
November 2021	3 (23.07%)	3.42 (0.72, 24.70)	0.15
December 2021	1 (7.70%)	4.14 (0.91, 29.43)	0.091
January 2022	2 (15.38%)	5.76 (1.33, 40.06)	0.034
February 2022	4 (30.76%)	8.72 (2.11, 59.74)	0.007
March 2022	0	8.72 (2.11, 59.74)	0.007
**Sex**			
Male	2 (15.38%)	2.41 (0.91, 5.78)	0.057
Female	11 (84.61%)		
Ticks			
Infested	9 (69.23%)	1.12 (0.64, 1.91)	0.680
Not infested	4 (30.76%)		

Legends: * indicate months of the rainy seasons.

## Data Availability

Not applicable.
